# Dual FDG/PSMA PET imaging to predict lesion-based progression of mCRPC during PSMA-RLT

**DOI:** 10.1038/s41598-024-61961-z

**Published:** 2024-05-17

**Authors:** Florian Rosar, Caroline Burgard, Scott David, Robert J. Marlowe, Mark Bartholomä, Stephan Maus, Sven Petto, Fadi Khreish, Andrea Schaefer-Schuler, Samer Ezziddin

**Affiliations:** 1 Departments of Nuclear Medicine, Saarland University – Medical Center, Kirrberger Str. 100, Geb. 50, 66421 Homburg, Germany; 2 Spencer-Fontayne Corporation, Jersey City, NJ USA

**Keywords:** mCRPC, PSMA, FDG, Mismatch, Radioligand therapy, Prostate cancer, Molecular medicine, Predictive markers

## Abstract

Candidates for prostate-specific membrane antigen (PSMA)-targeted radioligand therapy (RLT) of metastatic castration-resistant prostate cancer (mCRPC) frequently have “mismatch” lesions with pronounced 18-fluorodeoxyglucose ([^18^F]FDG) but attenuated PSMA ligand uptake on positron emission tomography (PET). However, no quantitative criteria yet exist to identify mismatch lesions and predict their response to RLT. To define such criteria, we retrospectively analyzed 267 randomly-selected glucometabolic mCRPC metastases from 22 patients. On baseline PET, we determined [^18^F]FDG and [^68^Ga]Ga-PSMA-11 maximum standardized uptake value (SUV_max_), and calculated the [^18^F]FDG SUV_max_/[^68^Ga]Ga-PSMA-11 SUV_max_ quotient (FPQ). From follow-up [^18^F]FDG PET after two lutetium-177-PSMA-617 RLT cycles, we evaluated the treatment response and categorized the lesions into three subgroups (partial remission, stable disease, progression) based on change in [^18^F]FDG SUV_max_. Lastly, we compared the baseline PET variables in progressing versus non-progressing lesions. Variables differing significantly, and a score incorporating them, were assessed via receiver operator characteristic (ROC) curve analysis, regarding ability to predict lesional progression, with area under the curve (AUC) as metric. Cut-offs with optimal sensitivity and specificity were determined using the maximum value of Youden's index. Fifty-one of 267 lesions (19.1%) progressed, 102/267 (38.2%) manifested stable disease, and 114/267 (42.7%) partially responded after two RLT cycles. At baseline, median [^68^Ga]Ga-PSMA-11 SUV_max_ was significantly lower (*p* < 0.001), median FPQ significantly higher (*p* < 0.001), and median [^18^F]FDG SUV_max_ similar in progressing versus non-progressing lesions. [^68^Ga]Ga-PSMA-11 SUV_max_ and FPQ showed predictive power regarding progression (AUCs: 0.89, 0.90). An introduced clinical score combining both further improved predictive performance (AUC: 0.94). Optimal cut-offs to foretell progression were: [^68^Ga]Ga-PSMA-11 SUV_max_ < 11.09 (88.2% sensitivity, 81.9% specificity), FPQ ≥ 0.92 (90.2% sensitivity, 78.7% specificity), clinical score ≥ 6/9 points (88.2% sensitivity, 87.5% specificity). At baseline, a low [^68^ Ga]Ga-PSMA-11 SUV_max_ and a high FPQ predict early lesional progression under RLT; [^18^F]FDG SUV_max_ does not. A score combining [^68^ Ga]Ga-PSMA-11 SUV_max_ and FPQ predicts early lesional progression even more effectively and might therefore be useful to quantitatively identify mismatch lesions.

## Introduction

Radioligand therapy (RLT) targeted at prostate-specific membrane antigen (PSMA) has emerged, and gained regulatory approval, as a promising new option for palliation and/or increased survival for many patients with metastatic castration-resistant prostate cancer (mCRPC)^1–3^. Also a target for positron emission tomography (PET) imaging of prostate cancer^4–12^, PSMA is a transmembrane glycoprotein that is typically, but not invariably, overexpressed on mCRPC cells^13–15^. Ample tumoral expression of this target, established on PET, is, unsurprisingly, the major eligibility requirement for PSMA-directed RLT of mCRPC^1–3,16^.

Prostate cancer cells with an aggressive phenotype, especially mCRPC cells, also frequently abundantly express glucose transporter 1 (GLUT1) due to heightened dependence on glucose intake and aerobic glycolysis for energy as mutations and tumor burden increase^17,18^. As on many other malignant cell types, GLUT1 overexpression on prostate cancer cells is reflected by uptake of the fluorine-18-conjugated glucose analogue fluorodeoxyglucose ([^18^F]FDG), shown on PET^19^. Higher [^18^F]FDG uptake now is well-recognized to correlate with prognostically-unfavorable pathological factors in prostate cancer in general^20,21^, and with poor outcomes in men receiving systemic treatment of mCRPC, including PSMA-targeted RLT^18,22–25^. As a result, [^18^F]FDG PET/computed tomography (CT) increasingly is being applied in mCRPC, to provide prognostic and/or predictive data^3,16,26–29^.

mCRPC lesions with high GLUT1 expression but attenuated or absent PSMA expression, as evidenced by uptake of the respectively-targeted radiotracers on PET, are termed “mismatch” lesions^30^. Due to the perceived reduced efficacy of RLT in mismatch lesions, the presence of such findings, indeed, sometimes even of a single mismatch lesion^31^, has been used as an exclusion criterion for PSMA-targeted RLT^32–34^. However, no quantitative uptake-related criteria based on both [^18^F]FDG PET and PSMA-targeted PET exist yet to identify such lesions and to predict their response to RLT. We therefore performed a retrospective analysis of mCRPC metastases seeking to use quantitative PET variables to identify PSMA ligand/[^18^F]FDG uptake profiles that predict RLT-refractory/resistant mCRPC lesions. Such characterization of mismatch lesions potentially will aid treatment-related decision-making in candidates for PSMA-targeted RLT.

## Materials and methods

### Endpoints

The first endpoint was each lesion’s uptake of [^68^Ga]Ga-PSMA-11 and of [^18^F]FDG, reflected by the maximum standardized uptake value (SUV_max_) of each radiotracer, on PET scans acquired before [^177^Lu]Lu-PSMA-617 RLT, i.e. at baseline. As part of this endpoint, we also calculated the pre-RLT [^18^F]FDG SUV_max_/[^68^Ga]Ga-PSMA-11 SUV_max_ quotient (FPQ) for each individual lesion.

The second endpoint was the evaluation of each lesion's response to two RLT cycles, based on changes in the [^18^F]FDG SUV_max_ between the baseline [^18^F]FDG PET/CT scan and a follow-up [^18^F]FDG scan acquired after the second cycle.

The third endpoint was the comparison of baseline [^68^Ga]Ga-PSMA-11 SUV_max_, baseline [^18^F]FDG SUV_max_, and baseline FPQ in the subgroup of progressing lesions versus the subgroup of lesions with disease stability or partial response after the two RLT administrations, and determination of adequate cut-off values of these variables to predict lesional outcome.

The last endpoint, based on these comparisons, was the development and preliminary assessment of a score to use baseline quantitative PET variables to predict whether a lesion would progress early in the course of RLT.

### Lesions, patients, and ethics

**Figure 1:**
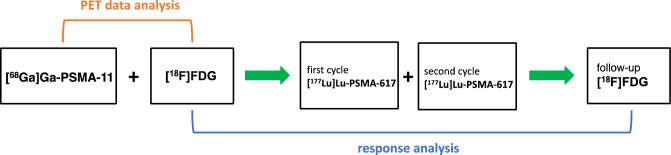
Schematic summary of study design.

The analysis included a sample of [^18^F]FDG-positive lesions in consecutive eligible patients with mCRPC who received two cycles of [^177^Lu]Lu-PSMA-617 RLT. Eligible patients had to have available PET imaging data from three scans: (1) baseline (pre-RLT) [^68^Ga]Ga-PSMA-11 PET/CT, (2) baseline [^18^F]FDG PET/CT, and (3) follow-up [^18^F]FDG PET/CT acquired shortly after completion of the two RLT cycles (Fig. 1). All patients received [^177^Lu]Lu-PSMA-617 RLT within a prospective patient registry (REALITY Study, NCT04833517). The analyzed imaging and treatment took place at the Saarland University Medical Center between 21 January 2019 and 21 December 2022. The selection of [^18^F]FDG-positive target lesions was random and did not consider uptake in PSMA ligand PET/CT.

A total of 267 lesions from 22 men (up to 14 per patient) were included in this analysis. Table 1 shows characteristics of the lesions and of the study sample. The analyzed lesions were mostly (almost 80%) in the skeleton. The patients tended to have late-stage or end-stage disease and were heavily pretreated. They received the PSMA-targeted RLT on a compassionate use basis according to §13 (2b) of the German Pharmaceutical Act. Patients gave written informed consent, which also covered participation in the registry and permission for de-identified patient data to be published in scientific communications. The study was approved by the local institutional review board (ethics committee approval number 140/17, 13 July 2017).

**Table 1:** Characteristics of the patients cohort and target lesions.

Characteristic	Value
Age [yr]	71 (55–84)
PSA at baseline [ng/mL], median (minimum–maximum)	469 (0.08–2907)
ECOG performance status category at baseline, % (n)	
0	14% (3)
1	45% (10)
≥ 2	41% (9)
Prior treatment, % [no.]	
Prostatectomy	55% (12)
Radiation therapy	64% (14)
ADT	100% (22)
NAAD	100% (22)
Chemotherapy	
Any	77% (17)
Docetaxel	77% (17)
Cabazitaxel	45% (10)
Other	14% (3)
Radium-223	14% (3)
[^177^Lu]Lu-PSMA-617 RLT activities [GBq], median (minimum–maximum)	
First activity	8.6 (1.1–10.6)
Second activity	8.2 (1.5–9.3)
Cumulative activity	16.7 (2.6–19.4)
Target lesions category, n (%)	
Any	267 (100%)
Lymph node	49 (18.4%)
Bone	212 (79.4%)
Liver	6 (2.2%)
Number per patient	
Mean ± SD	12 ± 4
Median (minimum–maximum)	14 (1–14)

*ADT* androgen deprivation therapy, *ECOG* eastern cooperative oncology group, *NAAD* novel androgen access drugs, *PSA*, prostate-specific antigen.

### PET/CT

Initial [^68^Ga]Ga-PSMA-11 PET/CT and [^18^F]FDG PET/CT were performed a mean ± standard deviation (SD) 25 ± 23 d and 18 ± 22 d, respectively, before PSMA-RLT started. The mean time between baseline [^68^Ga]Ga-PSMA-11 and baseline [^18^F]FDG PET/CT was 6 ± 8 d. The follow-up [^18^F]FDG PET/CT, acquired after two cycles PSMA-RLT, took place 122 ± 65 d after the baseline [^18^F]FDG PET/CT scan. Both [^68^Ga]Ga-PSMA-11 PET/CT and [^18^F]FDG PET/CT were performed ~ 60 min after intravenous injection of the radiotracer and a subsequent 500-mL infusion of NaCl 0.9%. A mean ± SD 142 ± 23 MBq of [^68^Ga]Ga-PSMA-11, 246 ± 42 MBq of [^18^F]FDG (baseline scan), and 259 ± 35 MBq of [^18^F]FDG (follow-up scan) were given. Patients fasted at least 4 h before each [^18^F]FDG infusion, and were instructed to void shortly before all PET/CT scans. Imaging was carried out applying standard protocols^35,36^. Whole-body PET images were acquired from vertex to mid-femur, using 3 min ([^68^Ga]Ga-PSMA-11) or 2 min ([^18^F]FDG) per bed position, with a 21.4-cm extended field-of-view. A Biograph 40 mCT scanner (Siemens Medical Solutions, Knoxville, TN, USA) was employed. For attenuation correction and anatomical localization*,* low-dose CT was performed together with the PET, using a 120-keV X-ray tube voltage. The tube current was modulated with CARE Dose4D software (Siemens Healthineers, Erlangen, Germany), with 30 mAs as the reference. PET datasets were reconstructed with an iterative 3-dimensional ordered-subset expectation maximization algorithm (3 iterations, 24 subsets) with gaussian filtering (5 mm full width at half maximum) and a 3 mm slice thickness. Besides attenuation correction, random, decay, and scatter correction were done.

### PET/CT data analysis

Firstly, [^18^F]FDG-positive target lesions of mCRPC were randomly selected and analyzed at baseline [^18^F]FDG PET/CT. Subsequently, the same target lesions were analyzed on the baseline [^68^Ga]Ga-PSMA-11 PET/CT scan. Care was taken to exclude foci representing characteristic inflammatory changes on [^18^F]FDG PET/CT, e.g. pulmonary inflammatory changes or reactive-inflammatory lymph nodes. For each target lesion and both radiotracer SUV_max_ was quantified, applying Syngo.via (Siemens Healthineers, Erlangen, Germany). In addition, FPQ was calculated for each individual lesion by dividing SUV_max_ of [^18^F]FDG by SUV_max_ of [^68^Ga]Ga-PSMA-11.

### RLT response classification

For each target lesion selected on baseline [^18^F]FDG PET/CT, SUV_max_ was analyzed on the follow-up scan after two cycles of [^177^Lu]Lu-PSMA-617 RLT. Response to the two cycles of [^177^Lu]Lu-PSMA-617 RLT was classified based on the change in lesional SUV_max_ (i.e., radiotracer uptake) from the baseline [^18^F]FDG scan to the follow-up [^18^F]FDG scan. Positron Emission Tomography Response Criteria in Solid Tumors (PERCIST)^37^ were used. According to these criteria, a ≥ 30% decrease from baseline SUV_max_ was defined as a partial response, a < 30% decrease to a < 30% increase, as stable disease, and a ≥ 30% increase, as progression.

### Statistics

Descriptive statistics are reported as mean ± SD, median (minimum–maximum), and/or number (percentage) or vice versa, as applicable. The Mann–Whitney-U test was used to compare baseline SUV_max_ of each radiotracer and baseline FPQ between the subgroup of lesions progressing after RLT versus the subgroup of non-progressing lesions, i.e. those exhibiting stable disease or partial response. Statistical significance was set at *p* < 0.05. All statistical analyses were carried out using Prism version 8 (GraphPad Software, San Diego, CA, USA). Variables showing a significant difference between lesion response subgroups were used to develop a lesion response prediction score for clinical application. For this score, points were assigned for different values of the respective variables with the goal of achieving a stepwise increment in lesional progression risk as the score increased.

Receiver operator characteristic (ROC) curve analysis was carried out to assess predictive performance of the significant variables and of the score. The perfomance metric was the area under the ROC curve (AUC). For each variable and for the predictive score, the maximum value of the Youden's index (*J*) was used to determine the cut-off that attained optimal sensitivity and specificity.

### Ethics approval and consent to participate

All procedures performed in the patients described herein were in accordance with the ethical standards of the Institutional and/or National Research Ethics Committees and with the 1964 Helsinki Declaration and its later amendments, or with comparable ethical standards. This analysis was approved by the Institutional Review Board of the Ärztekammer des Saarlandes/Saarbrücken (approval number: 140/17, approval date: 13 July 2017. This report does not include any animal studies. Written informed consent was obtained from all participants.

## Results

**Figure 2:**
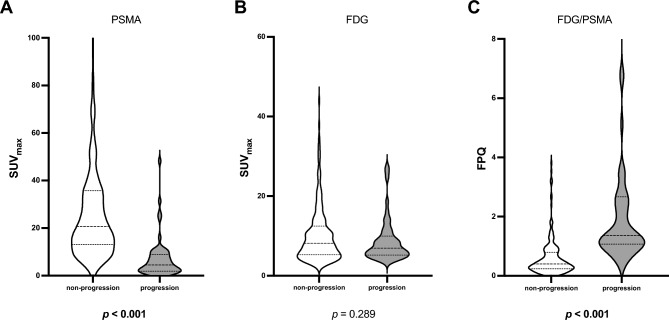
Baseline PET variables by lesional response to 2 cycles of [^177^Lu]Lu-PSMA-617 RLT: (**A**) SUV_max_ on [^68^Ga]Ga-PSMA-11 PET/CT, (**B**) SUV_max_ on [^18^F]FDG PET/CT, and (**C**) FPQ, [^18^F]FDG **SUV**_**max**_**/**[^68^Ga]Ga-PSMA-11 **SUV**_**max**_ quotient.

**Figure 3:**
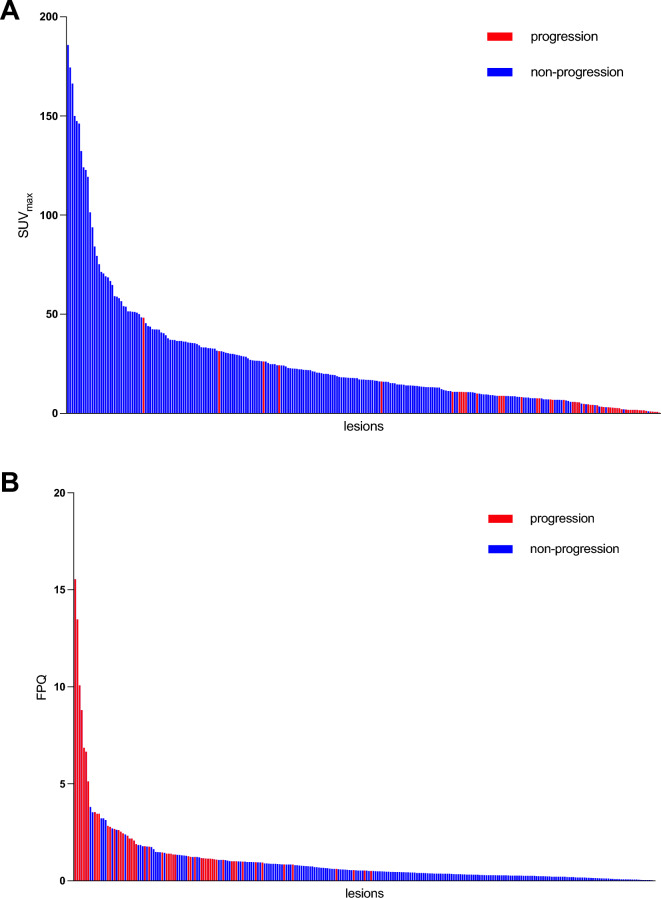
Waterfall plots showing lesional response to 2 cycles of [^177^Lu]Lu-PSMA-617 RLT by value of the lesional (**A**) SUV_max_ on the baseline [^68^Ga]Ga-PSMA-11 PET/CT and (**B**) FPQ, [^18^F]FDG **SUV**_**max**_**/**[^68^Ga]Ga-PSMA-11 **SUV**_**max**_ quotient.

**Figure 4:**
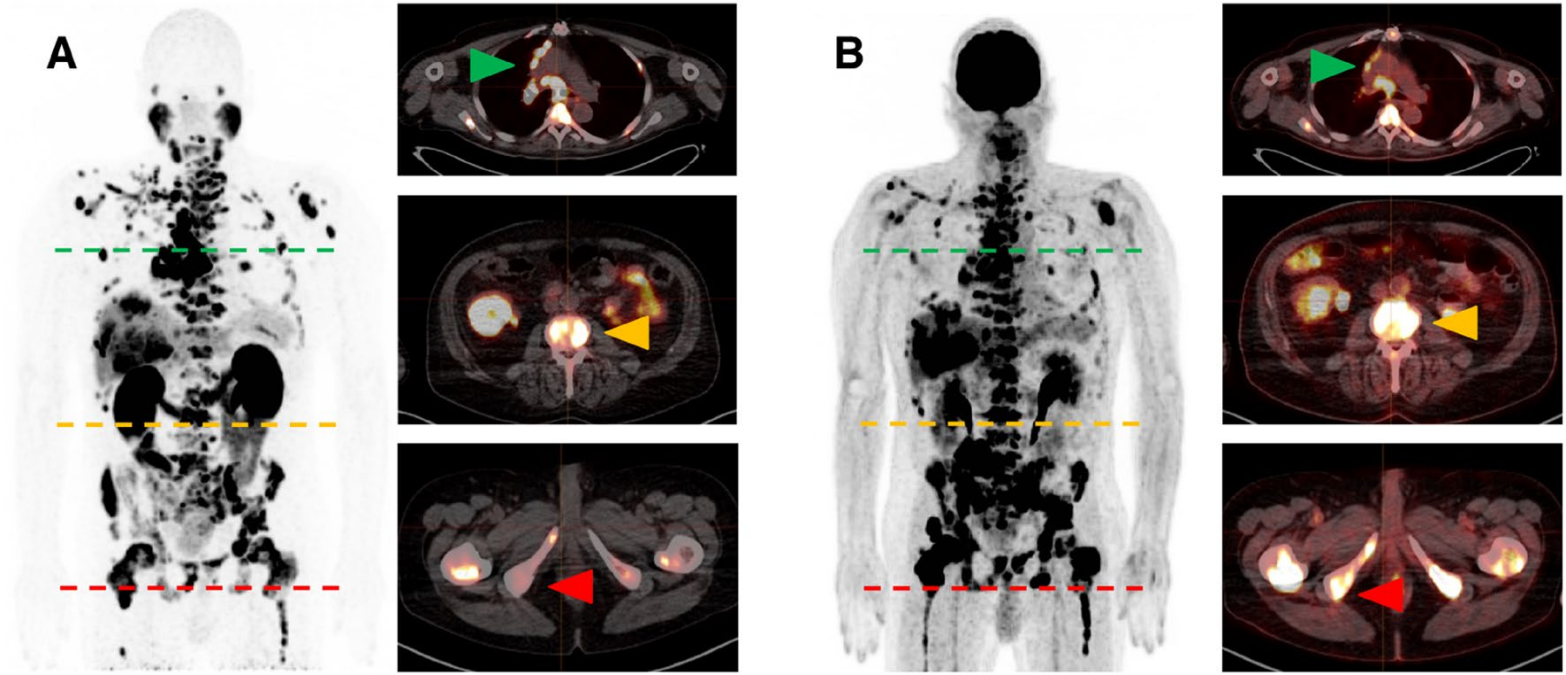
Representative whole-body and transversal slice images from (**A**) baseline [^68^Ga]Ga-PSMA-11 PET/CT scan and (**B**) baseline [^18^F]FDG-PET/CT scan of a mCRPC patient showing different patterns of [^68^Ga]Ga-PSMA-11 uptake and [^18^F]FDG uptake in different lesions. Green arrows denote lymph node metastases with intense [^68^Ga]Ga-PSMA-11 and moderate [^18^F]FDG uptake. Yellow arrows indicate bone metastases with intense uptake of both radiotracers. Red arrows indicate bone metastases with faint [^68^Ga]Ga-PSMA-11 uptake but intense [^18^F]FDG uptake.

Lesional values were highly variable for [^68^Ga]Ga-PSMA-11 SUV_max_, [^18^F]FDG SUV_max_, and FPQ, as reflected by large SDs relative to mean values and by wide minimum–maximum ranges (Table 2, Figs. 2 and 3). At baseline, 72/267 lesions (27.0%) had greater [^18^F]FDG uptake than [^68^Ga]Ga-PSMA-11 uptake i.e. an FPQ > 1, and 195/267 lesions (73.0%), equal or lesser [^18^F]FDG uptake than [^68^Ga]Ga-PSMA-11 uptake, i.e. an FPQ ≤ 1. Representative images of lesions showing different patterns of [^18^F]FDG and [^68^Ga]Ga-PSMA-11 uptake are seen in Fig. 4.

**Table 2:** PET variables of target lesions at baseline.

Characteristic/finding	Value		
Lesional SUV_max_			
Baseline [^68^Ga]Ga-PSMA-11 PET/CT			
Median (minimum–maximum)	17.03 (0.35–185.83)		
Mean ± SD	26.12 ± 29.98		
Baseline [^18^F]FDG PET/CT			
Median (minimum–maximum)	7.94 (2.28–44.00)		
Mean ± SD	9.95 ± 6.84		
Baseline FPQ			
Median (minimum–maximum)	0.51 (0.03–15.54)		
Mean ± SD	0.99 ± 1.69		
Category, % of study sample (n)			
≤ 1	73.0% (195/267)		
> 1	27.0% (72/267)		
Lesional response^a^ to RLT, % of study sample (n)			
Partial response	42.7% (114/267)		
Stable disease	38.2% (102/267)		
Progression	19.1% (51/267)		

	Non-progressive lesions (n = 216)^b^	Progressive lesions (n = 51)	*p*
SUV_max_, baseline [^68^Ga]Ga-PSMA-11 PET/CT			
Median (minimum–maximum)	20.77 (1.03–185.80)	4.48 (0.35–48.25)	** < 0.001**
Mean ± SD	30.59 ± 31.45	7.19 ± 8.68	
SUV_max_, baseline [^18^F]FDG PET/CT			
Median (minimum–maximum)	8.15 (2.28–44.00)	6.90 (3.69–27.37)	0.289
Mean ± SD	10.22 ± 7.11	8.79 ± 5.47	
FPQ, baseline PET/CT scans			
Median (minimum–maximum)	0.40 (0.03–3.81)	1.36 (0.51–15.54)	** < 0.001**
Mean ± SD	0.60 ± 0.63	2.65 ± 3.16	

^*18*^*F* fluorine-18, ^*68*^*Ga* gallium-68, *CT* computed tomography, *FDG* fluorodeoxyglucose, *FPQ* [^18^F]FDG/[^68^Ga]Ga-PSMA-11 SUV_max_ quotient, *PET* positron emission tomography, *PSMA* prostate-specific membrane antigen, *RLT* radioligand therapy, *SD* standard deviation, *SUV*_*max*_ maximum standardized uptake value.

*p* values in bold were statistically significant at *p* < 0.05.

^a^Lesional response classifications are based on change from baseline to post-2nd cycle of RLT in [^18^F]FDG SUV_max_, utilizing PERCIST 1.0 criteria^37^.

^b^“Non-progressing lesions” include those showing partial response (n = 114, 52.8% of non-progressing lesions) or stable disease (n = 102, 47.2% of non-progressing lesions).

**Figure 5:**
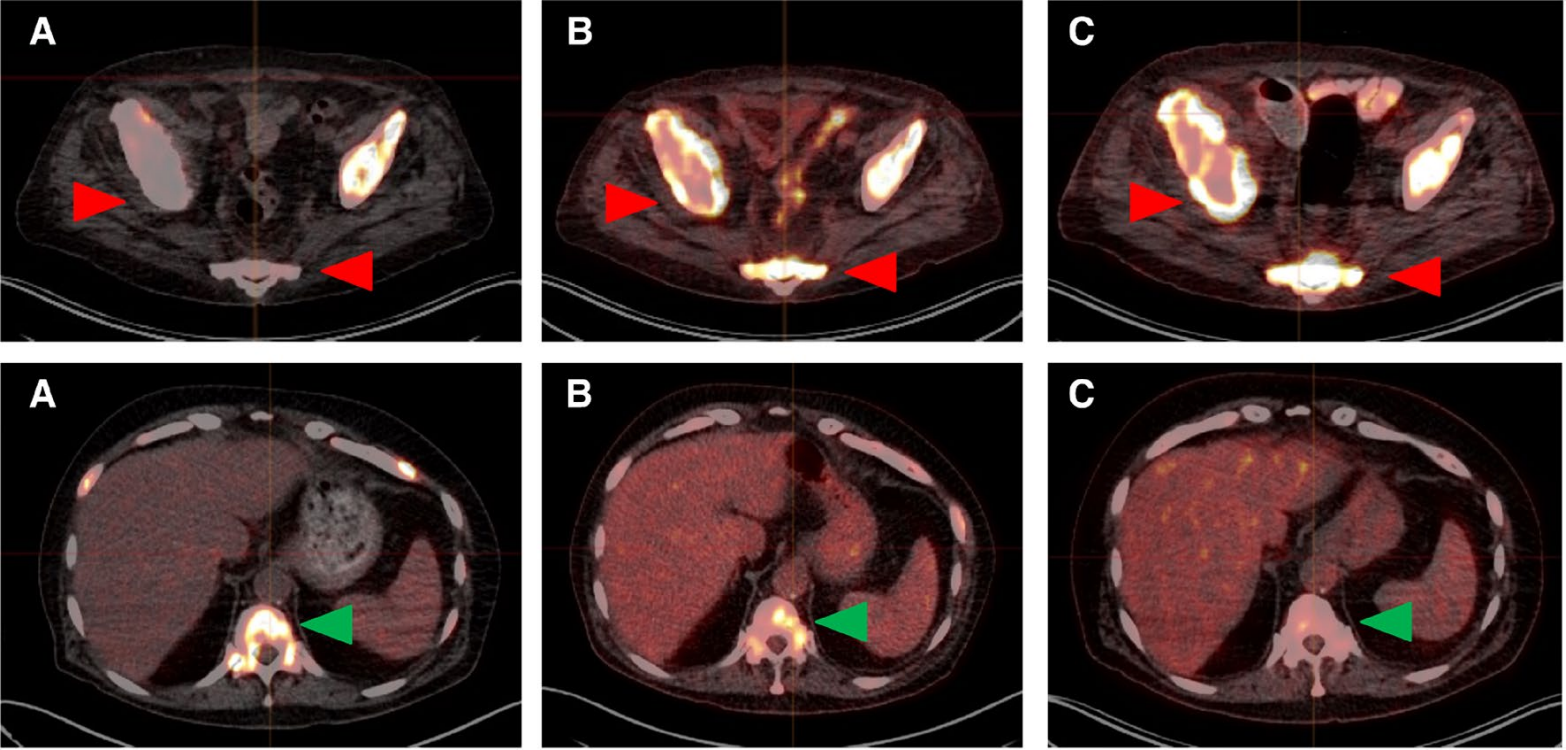
Representative transverse slice images showing lesion-based progression (first row) and partial response (second row) to 2 cycles of [^177^Lu]Lu-PSMA-617 RLT: (**A**) baseline [^68^Ga]Ga-PSMA-11 PET/CT, (**B**) baseline [^18^F]FDG PET/CT, and (**C**) follow-up [^18^F]FDG PET/CT. First row, red arrows: progressing bone metastases. These lesions had faint [^68^Ga]Ga-PSMA-11 uptake (SUV_max_: 3.47 and 4.00, respectively) and intense [^18^F]FDG uptake (SUV_max_: 11.17 and 8.67, respectively) in the baseline scans (FPQ: 3.22 and 2.17, respectively), and increased [^18^F]FDG uptake (SUV_max_ 16.87 and 12.37, respectively; 51% higher and 43% higher, respectively, than at baseline) in the follow-up scan. Second row, green arrows: bone metastasis showing partial response. The lesion, which had intense [^68^Ga]Ga-PSMA-11 uptake (SUV_max_: 22.74) and moderate [^18^F]FDG uptake (SUV_max_: 6.60) in the respective baseline scans (FPQ: 0.29), had decreased [^18^F]FDG uptake (SUV_max_ 4.52, 32% lower than at baseline) in the follow-up scan.

After two cycles of [^177^Lu]Lu-PSMA-617 RLT, relatively few lesions showed PERCIST progression pertaining to glucose metabolism: [^18^F]FDG SUV_max_ increased ≥ 30% from baseline in 51/267 (19.1%) (Table 2). Of non-progressing lesions, a slight majority showed partial response (114/267, 42.7% of all analyzed lesions) versus stable disease (102/267, 38.2% of all analyzed lesions). Representative images of lesional responses are seen in Fig. 5.

At baseline, [^68^Ga]Ga-PSMA-11 SUV_max_ was significantly lower in lesions that went on to progress after two RLT cycles than in lesions that went on to show stable disease or partial response (7.19 ± 8.68 vs. 30.59 ± 31.45, *p* < 0.001). Conversely, baseline [^18^F]FDG SUV_max_ did not differ between these subgroups (8.79 ± 5.47 vs. 10.22 ± 7.11, *p* = 0.29) (Fig. 2A and B). However, the FPQ, i.e. the ratio of [^18^F]FDG uptake to [^68^Ga]Ga-PSMA-11 uptake, in progressing lesions significantly exceeded that in non-progressing lesions (2.65 ± 3.16 vs. 0.60 ± 0.63,* p* < 0.001) (Fig. 2C). Figure 3 depicts as waterfall plots the lesions’ individual values for baseline [^68^Ga]Ga-PSMA-11 SUV_max_ and baseline FPQ. No lesion with a baseline [^68^Ga]Ga-PSMA-11 SUV_max_ ≥ 50 or a baseline FPQ < 0.5 showed [^18^F]FDG metabolic imaging progression.

**Figure 6:**
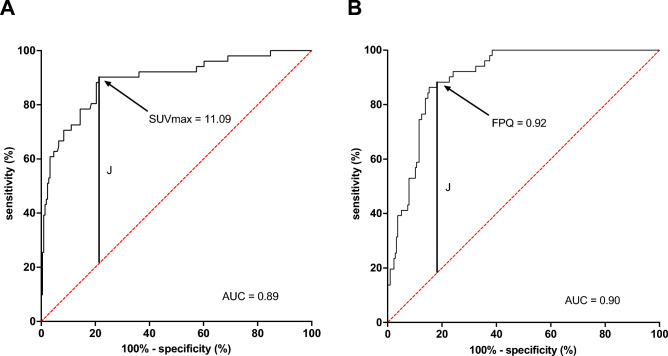
ROC curves showing the performance to predict lesional progression of (**A**) lesional SUV_max_ on the baseline [^68^Ga]Ga-PSMA-11 PET/CT scan or (**B**) FPQ, [^18^F]FDG **SUV**_**max**_** /**[^68^Ga]Ga-PSMA-11 **SUV**_**max**_ quotient. The respective maximum values (*J*) of the Youden's index identified a [^68^Ga]Ga-PSMA-11 SUV_max_ of < 11.09 (sensitivity 88.2%, specificity 81.9%) or an FPQ of ≥ 0.92 (sensitivity 90.2%, specificity 78.7%) as optimal threshholds.

On ROC curve analysis, baseline [^68^Ga]Ga-PSMA-11 SUV_max_ and baseline FPQ showed similar performance to discriminate between lesions that would progress after two RLT cycles versus lesions that would not: respective AUCs were 0.89 versus 0.90. The respective maximum values of the Youden's index (*J*) identified a baseline [^68^Ga]Ga-PSMA-11 SUV_max_ of < 11.09 (Fig. 6A) or a baseline FPQ of ≥ 0.92 (Fig. 6B) as the threshold values of these variables that had optimal sensitivity and specificity in distinguishing lesional progression versus non-progression. At these thresholds, the two variables again showed comparable performance: respectively, 88.2% sensitivity and 81.9% specificity (OR 34.0), versus 90.2% sensitivity and 78.7% specificity (OR 34.0).

**Figure 7:**
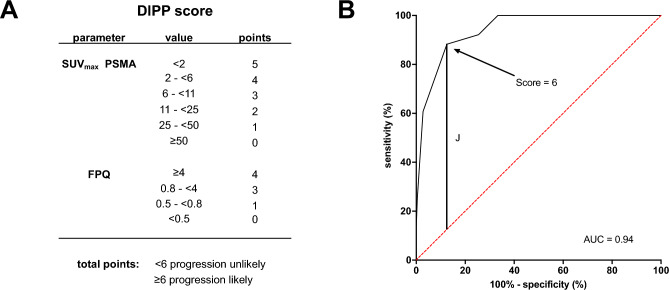
(**A**) Dual imaging progression prediction (DIPP) score and (**B**) ROC curve showing the score’s performance to predict lesional progression. The maximum value of the Youden’s index (*J*) identified a DIPP score of ≥ 6 on a 0–9-point scale (sensitivity 88.2%, specificity 87.5%) as showing optimal predictive performance among DIPP score values.

A clinical score incorporating the two variables, the dual imaging progression prediction (DIPP) score, was developed (Fig. 7A). Using ROC curve analysis, the DIPP score achieved an AUC of 0.94 to predict lesional response (Fig. 7B). The maximum value of the Youden's index (*J*) determined a DIPP score ≥ 6 on the 0–9-point scale to be the optimal threshold denoting high risk of lesional progression. With 88.2% sensitivity and 87.5% specificity (OR 52.5), this DIPP score threshold appeared to have comparable sensitivity, but appreciably improved specificity, relative to each of its component variables at their optimal thresholds.

## Discussion

This study is, to our knowledge, the first yet published that addresses the unmet need for lesion-based quantification of mismatch, by analyzing the relationship of baseline [^68^Ga]Ga-PSMA-11 and baseline [^18^F]FDG PET variables to RLT response, and introducing a scoring system that appears to effectively predict early lesional progression.

Our key finding was that efficient prediction of lesional response to RLT may be provided by considering not just the "traditional" eligibility criterion of PSMA ligand uptake, i.e. SUV_max_, but an additional PET variable, the FPQ, i.e. the ratio of [^18^F]FDG SUV_max_ to [^68^Ga]Ga-PSMA-11 SUV_max_. Even better prediction may be achieved by using the DIPP score introduced here, which combines these two variables, and which can be easily and quickly calculated.

Our observation that the relationship of [^18^F]FDG SUV_max_ to [^68^Ga]Ga-PSMA-11 SUV_max_ i.e. the FPQ, predicted lesional response to PSMA-targeted RLT, aligns with the findings of a number of studies suggesting predictive and prognostic power in prostate cancer of the [^18^F]FDG SUV_max_ of a lesion or of the total tumor burden. These studies showed these variables to be associated with negative disease characteristics and poor patient outcomes^19–21,38–40^, including in men with mCRPC^18,22,41,42^, and those undergoing PSMA-targeted RLT^25,29^. Thus, our analysis furnishes additional evidence buttressing use of [^18^F]FDG PET/CT in screening candidates for PSMA-targeted RLT.

However, our study also supplies evidence supporting the (unsurprising) primacy of PSMA ligand uptake in predicting lesional response to PSMA-targeted RLT. We noted that baseline [^18^F]FDG SUV_max_ alone did not differ between progressing and non-progressing lesions. By contrast, baseline [^68^Ga]Ga-PSMA-11 SUV_max_ was a strong predictor of lesional progression to PSMA-targeted RLT both by itself (at a cut-off of < 11.09, 88.2% sensitivity and 81.9% specificity) and as part of the FPQ (at a cut-off of ≥ 0.92, 90.2% sensitivity and 78.7% specificity). Moreover, considering PSMA ligand uptake as an absolute value as well as a relative value (i.e. as the denominator of the FPQ), combined into the DIPP score, further strengthened predictive performance (at a cut-off of ≥ 6/9 points, 88.2% sensitivity and 87.5% specificity).

Also of interest, there was an 80% rate of lesional stable disease or partial response in our study, notwithstanding the high degree of glucometabolism in many of the analyzed lesions. These observations support the notion that RLT may be beneficial even in lesions with high [^18^F]FDG uptake, so long as a sufficient proportion of cells has adequate PSMA expression—a factor that may be readily discerned using the FPQ. The benefit of RLT in such cases also has been suggested by others^29,43^.

A routine implementation of [^18^F]FDG PET/CT in clinical practice to screen candidates for PSMA-targeted RLT certainly brings additional information compared with a (contrast-enhanced) diagnostic CT but may have to be weighed against the potential financial burden regarding additional costs.

Based on our data, a mismatch lesion with insufficient PSMA expression and potential resistance to PSMA-targeted RLT can be identified by using the variables PSMA PET-derived SUVmax and FPQ. Compared to [^68^Ga]Ga-PSMA-11 SUV_max_ or FPQ alone, a score combining both further strengthens predictive performance. We propose that a mismatch finding can be identified with a DIPP score of ≥ 6. Quantitatively indentifying mismatch lesions may facilitate individualized decision-making regarding PSMA-targeted RLT and the design of individualized mCRPC management strategies. However, the feasibility of implementing the DIPP score into clinical routine needs to be evaluated. We recommend studies, ideally prospective, in larger patient cohorts verifying our observations and addressing the clinical impact of this approach.

This analysis has several limitations. Firstly, the retrospective, single-center study design may impose bias and decrease generalizability and transferability. Other cutoff values would be expected for the use of different PSMA ligands or other reconstruction settings. Secondly, a possible selection bias cannot be excluded, as only a (pre-)selected number of patients who participated in the REALITY study received baseline and follow-up [^18^F]FDG PET/CT. The results of the present study should be treated with caution when compared with outcome data from other studies as our criterion of lesional progression was based on [^18^F]FDG PET/CT which is not established for prostate cancer such as response based on PSMA PET/CT or structural changes as reflected by Response Criteria in Solid Tumors^44^. Furthermore, our definition of progression was formulated on a lesional basis, not a patient basis. Further studies should be undertaken to consider relationships of PSMA ligand uptake and [^18^F]FDG uptake across the total tumor burden, e.g. in form of whole-body PET variables such as total lesion PSMA and total lesion glycolysis. These variables should be analyzed for their association with patient-based RLT response or overall survival. Lastly, we did not validate the DIPP score in a sample of lesions separate from those used to develop the score. The score's predictive performance should be confirmed in this way.

## Conclusions

A low baseline SUV_max_ on [^68^Ga]Ga-PSMA-11 PET/CT and high baseline FPQ, which incorporates the former as well as baseline [^18^F]FDG SUV_max_, predict lesional progression early in the course of RLT, whereas baseline [^18^F]FDG SUV_max_ by itself does not. Compared to [^68^Ga]Ga-PSMA-11 SUV_max_ or FPQ alone, a score combining both predicts early lesional progression even more effectively and might therefore be useful to quantitatively identify mismatch lesions.

## Data Availability

The datasets used and analyzed during the current study are available from the corresponding author on reasonable request.

## Abbreviations

^18^F: Fluorine-18
^68^ Ga: Gallium-68
^177^Lu: Lutetium-177
ADT: Androgen deprivation therapy
AUC: Area under the receiver operator characteristic curve
CT: Computed tomography
DIPP: Dual imaging progression prediction
ECOG: Eastern cooperative oncology group
FDG: Fluorodeoxyglucose
NAAD: Novel androgen axis drugs
PERCIST: Positron emission tomography response criteria in solid tumors
PET: Positron emission tomography
p.i.: Post-injection
PSA: Prostate-specific antigen
PSMA: Prostate-specific membrane antigen
RLT: Radioligand therapy
ROC: Receiver operator characteristic
SD: Standard deviation
SUV_max_: Maximum standardized uptake value
